# Predicting the onset of chronic kidney disease (CKD) for diabetic patients with aggregated longitudinal EMR data

**DOI:** 10.1371/journal.pdig.0000700

**Published:** 2025-01-22

**Authors:** Neda Aminnejad, Michelle Greiver, Huaxiong Huang

**Affiliations:** 1 Department of Mathematics & Statistics, York University, Toronto, Canada; 2 Department of Family & Community Medicine, University of Toronto, Toronto, Canada; 3 Department of Family & Community Medicine, North York General Hospital, Toronto, Canada; Reader in Forensic Intelligent Data Analysis, UNITED KINGDOM OF GREAT BRITAIN AND NORTHERN IRELAND

## Abstract

Chronic kidney disease (CKD) affects over 13% of the population, totaling more than 800 million individuals worldwide. Timely identification and intervention are crucial to delay CKD progression and improve patient outcomes. This research focuses on developing a predictive model to classify diabetic patients showing signs of kidney function impairment based on their CKD development risk. Our model utilizes electronic medical record (EMR) data, specifically by incorporating patient demographics, laboratory results, chronic conditions, risk factors, and medication codes to predict the onset of CKD in diabetic patients six months in advance, achieving an average Area Under the Curve (AUC) of 0.88. We leverage aggregated EMR data to effectively capture relevant information within the observation year instead of using temporal EMR data. Furthermore, we identify the most significant features for predicting CKD onset, including mean, minimum, and first quartile of estimated glomerular filtration rate (eGFR) during the observation year, along with variables such as diagnosis age and duration of hypertension, osteoarthritis, and diabetes, as well as levels of hemoglobin and fasting blood glucose (FBG). We also explored a refined model utilizing only these most significant features, which yields a slightly lower AUC of 0.86. These variables are typically available in primary data, empowering physicians for real-time risk assessment. The proposed model’s ability to identify higher-risk patients is essential for timely intervention, personalized care, risk stratification, patient education, and potential cost savings. This research contributes valuable insights for healthcare practitioners seeking efficient tools for early CKD detection in diabetic populations.

## Author summary

This study introduces a predictive model for identifying chronic kidney disease (CKD) onset in diabetic patients using aggregated electronic medical record (EMR) data. With CKD affecting over 800 million people globally, early detection is crucial for disease management. The model, incorporating demographics, lab results, and chronic condition histories, predicts CKD onset six months in advance with an AUC of 0.88. Key predictors include eGFR metrics, age at diagnosis, and duration of comorbidities like hypertension and diabetes. A refined version using only significant features achieved an AUC of 0.86, showcasing its efficiency. This model holds promise for real-time risk assessment and timely interventions in healthcare, though further validation across diverse populations is needed.

## Introduction

Chronic kidney disease (CKD) is a complication that commonly occurs in diabetic patients. Current research estimates that CKD impacts approximately 843.6 million individuals worldwide [1]. CKD stages are defined by the estimated glomerular filtration rate (eGFR) measuring kidney function and span from normal (Stage 1) to end-stage renal disease (Stage 5), where kidney function has drastically declined or is lost. A study in 2016 that performed a systematic review and meta-analysis of 100 studies reported a global prevalence of CKD at 13.4%, with stages 3 to 5 at 10.6% [2]. In 2017, the global age-standardized prevalence of diabetes-related CKD was reported to be 15.48 per 1,000 men and 16.50 per 1,000 women [3]. In individuals with type 2 diabetes mellitus (T2DM), the prevalence of CKD varies globally, ranging from 27% in China to as high as 84% in Tanzania [4]. Additionally, it is anticipated that the burden of CKD will rise in the future, particularly as the world population ages and conditions such as hypertension, obesity, and T2DM become more prevalent [5]. In the United States, data from the National Health and Nutrition Examination Survey (NHANES) indicated an increase in the proportion of diabetic patients with CKD stages 3–4, rising from 20% between 1999 and 2004 to 25% between 2011 and 2014 [3]. Early detection, treatment, and management of CKD play a pivotal role in averting or delaying complications such as end-stage renal disease (ESRD), necessitating dialysis or transplantation. Notably, excess mortality associated with Type 1 and Type 2 Diabetes (T1DM and T2DM) is predominantly observed in individuals with CKD [6, 7]. Patients with diabetes-related CKD tend to have poorer survival rates compared to those without CKD, largely due to the higher risk of associated health complications [8]. CKD is linked to an eight- to ten-fold increase in the risk of cardiovascular mortality, acting as a significant risk amplifier in individuals with both diabetes and hypertension [9]. Consequently, the timely identification of CKD in diabetic patients is key for preventing or mitigating these complications and effectively managing CKD. Notably, diabetic kidney disease (DKD) is not the sole type of kidney disease affecting diabetic patients. Those with both CKD and diabetes may exhibit either DKD, where CKD results directly from diabetes, or nondiabetic kidney disease (NDKD) involving CKD induced by factors unrelated to diabetes. Some individuals may even experience a combination of both DKD and NDKD [10]. This study considers CKD regardless of DKD or NDKD subtypes.

**Fig 1:**
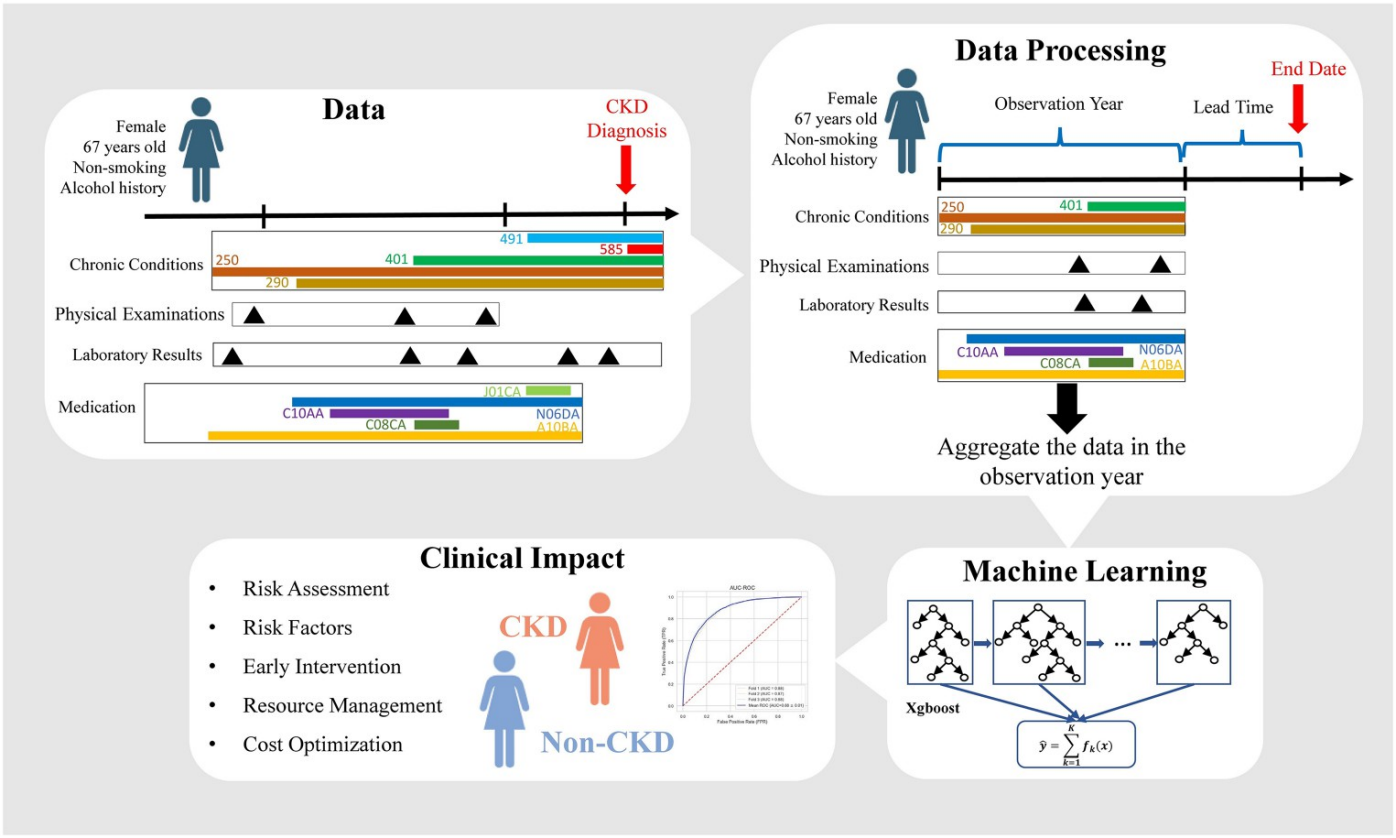
Schematic of the general framework of our proposed model. Each patient’s physical examinations, laboratory results, chronic condition codes, and medication codes are aggregated within the observation year and integrated with demographics and risk factors. Subsequently, this aggregated data is utilized as input for the Xgboost model to predict the CKD incidence in diabetic patients showing signs of kidney function impairment 6 months in advance.

There are various studies that employ healthcare data and machine learning to identify CKD. Low et al. [11] developed a stepwise multivariable logistic regression to identify baseline predictors for predicting the CKD incidence in the median follow-up of 5.5 years and achieved AUC of 0.83. Allen et al. [12] developed two machine learning algorithms to predict risk within 5 years for CKD development at the time of T2DM diagnosis, using 1 year of prior Electronic Health Record (EHR) data and achieved AUC of 0.83. Song et al. [13] built a Gradient Boosting Model based on 96, 605 distinct features as the baseline model to predict CKD onset in 6 months and achieved an AUC of 0.86 and evaluated how different levels of medication and diagnosis codes would affect prediction performance. Dong et al. [14] implemented seven machine learning algorithms based on medical characteristics available from EMR to predict the risk of developing CKD over the next 3 years and achieved the highest AUC of 0.815. In this study, we implement a machine learning model to predict CKD development in six months based on EMR primary care data from a cohort of diabetic patients showing signs of kidney function impairment. Fig 1 provides an overview of the proposed model. The highlights of our study are as follows:

- We utilize a cohort of diabetic patients, data elements include demographic information, chronic diseases, medication codes, risk factors, physical examinations, and laboratory results.
- We aggregate the irregular temporal data in the observation year using two different approaches. First, for the binary variables, such as chronic condition codes and medication codes, we aggregated the presence of these variables in the observation year. Second, we calculated 8 summary statistics of the values in the observation year to capture the variation of each laboratory result and physical examination.
- We employ the Xgboost machine-learning model to predict the onset of CKD six months in advance based on 786 variables.
- We identify and analyze the most significant features influencing CKD development in our prediction model.
- To investigate the applicability of the model, we explored the prediction power of two refined models, one with only eGFR summary statistics as input and one with only the most significant features as input.
- The proposed algorithm allows the flexibility to integrate various binary and continuous variables in EMR data without confronting time irregularity in EHR data.
- The developed risk stratification model could be integrated into healthcare management guidelines, promoting a more data-driven utilization of healthcare management resources.

## Methods and materials

### Data

The data was extracted from the Diabetes Action Canada (DAC) National Data Repository and consists of de-identified Electronic Medical Records (EMR) of diabetic patients in the Secure Analytic Virtual Environment (SAVE) with limited DAC-approved access. Data in the National Data Repository is collected through semi-annual extractions from consenting physicians at Practice-Based Research and Learning Networks across Canada who contribute data to the Canadian Primary Care Sentinel Surveillance Network (CPCSSN). For this study, data from patients with diabetes from five provinces was used with an age and sex-matched control group. During each extraction, records are cleaned, coded, and de-identified. Data are extracted from specific fields (e.g., billing, health condition, reason for visit, lab, medications). This dataset represents patients’ primary care (i.e., routine care) in Canada. It captures all primary care visits, excluding emergency room and specialist visits. All laboratory test results, including those ordered by specialists, are included. It is a comprehensive primary care data set for individual patients, but it comes with a caveat: since the data is mainly derived from Family Health Teams (FHT), patients from solo practitioners, community health centers, and walk-in clinics may not be well represented.

We excluded all patients with any manifestation of kidney disease prior to the observation year (e.g., CKD diagnosis code, dialysis, kidney transplant). Individuals in the cohort were identified as having CKD if they had two consecutive eGFR values of < 60ml/min per 1.73 *m*^2^ for more than 3 months apart. This definition was based on existing national and international CKD frameworks and conventions [15]. Table 1 presents the CKD stages defined by eGFR values calculated using the 2021 CKD-EPI formula [16, 17]

$$\begin{array}{c} \text{eGFR}=142\times min{\left(SCr/\kappa ,1\right)}^{\alpha }\times max{\left(SCr/\kappa ,1\right)}^{-1.200}\times 0.9938^{age}\times 1.012\;\left[\text{if}\;\text{female}\right] \end{array}$$

where *SCr* is serum creatinine, *κ* is 0.7 for females and 0.9 for males, *α* is -0.241 for females and -0.302 for males.

**Table 1:** Definition of CKD stages by eGFR.

Stages	eGFR (*mL*/*min*/1.73*m*^2^)	Terms
Stage 1	≥ 90	Normal
Stage 2	60–89	Mildly decreased
Stage 3a	45–59	Mildly to moderately decreased
Stage 3b	30–44	Moderately to severely decreased
Stage 4	15–29	Severely decreased
Stage 5	< 15	End-Stage Renal Disease (ESRD)

The table summarizes the CKD stages based on eGFR values. CKD onset is considered CKD stage 3a when a patient has two consecutive eGFR values of < 60 *mL*/*min*/1.73*m*^2^ for more than 3 months apart.

The available datasets include demographic information, laboratory results, physical examinations, risk factors, medication codes, and diagnosis codes for chronic conditions. Medications and diagnosis codes are presented in the data as ATC and ICD9 codes, respectively. To reduce the dimensionality, the granularity of medication and diagnosis codes is retained using only the first 5 and 3 characters, respectively. Table 2 summarizes the available variables in the study.

**Table 2:** Summary of available variables.

Category	Variables
Demographics	Age, Sex
Risk Factors	Alcohol History, Smoking History
Laboratory Results	A1c, FBG, HDLC, Hb, LDLc, SCr, TCH, TG, eGFR
Physical Examination	BMI, sBP
Diagnosis Codes of Chronic Condition	360 ICD9 codes
Medication Codes	314 ATC codes
Diagnosis Age and Duration	Duration, and Diagnosis Age of 9 Chronic Conditions (COPD, Dementia, Depression, Diabetes, Epilepsy, Herpes Zoster, Hypertension, Osteoarthritis, Parkinson’s)

The table summarizes the available dataset for this study. The granularity of medication and diagnosis codes is retained as the first 5 and 3 characters, respectively.

After pre-processing the data of over 48, 052 diabetic patients, 15, 638 diabetic patients were found to be eligible for our model, 20.3% of which developed CKD after six months. The average age of the cohort is 68.1 years, and 56.5% of the cohort is male. Hypertension emerges as the most prevalent comorbidity within the diabetic patients showing signs of kidney function impairment, affecting 65% of patients, followed by osteoarthritis at 30.3%. HMG CoA reductase inhibitors group (ATC C10AA) is the most consumed medication group in the observation year with a prevalence of 26%, followed by the Biguanides group (ATC A10BA) with a prevalence of 21.8%. Table 3 demonstrates the baseline characteristics of the cohort for this study.

**Table 3:** Baseline characteristics of the cohort.

	All Patients (n = 15,368)	CKD (n = 3,123)	non-CKD (12,245)
Age (mean)	68.1	70.9	67.4
Sex (Female)	6,687 (43.5%)	1,445 (46.3%)	5,242 (42.8%)
Alcohol History	3,599 (23.4%)	664 (21.3%)	2,935 (24%)
Smoking History	2,127 (13.8%)	395 (12.6%)	1,732 (14.1%)
Hypertension	9,988 (65%)	2,176 (69.7%)	7,812 (63.8%)
Osteoarthritis	4,663 (30.3%)	898 (28.8%)	3,765 (30.7%)
Depression	2,914 (19%)	487 (15.6%)	2,427 (19.8%)
Disorders of Lipoid Metabolism	1,596 (10.4%)	348 (11.1%)	1,248 (10.2%)
Chronic Bronchitis	1,547 (10.1%)	353 (11.3%)	1,194 (9.8%)
ATC C10AA	4,004 (26.1%)	866 (27.7%)	3,138 (25.6%)
ATC A10BA	3,351 (21.8%)	768 (24.6%)	2,583 (21.1%)
ATC C09AA	2,305 (15%)	550 (17.6%)	1,755 (14.3%)
ATC A02BC	1,911 (12.4%)	462 (14.8%)	1,449 (11.8%)
ATC C08CA	1,473 (9.6%)	408 (13.1%)	1,065 (8.7%)

The table summarizes age, sex, risk factors, and most prevalent chronic conditions and medications (diabetes and CKD are excluded since all patients have diabetes in the observation year and no patient has developed CKD in the observation year).

### Data processing

**Fig 2:**
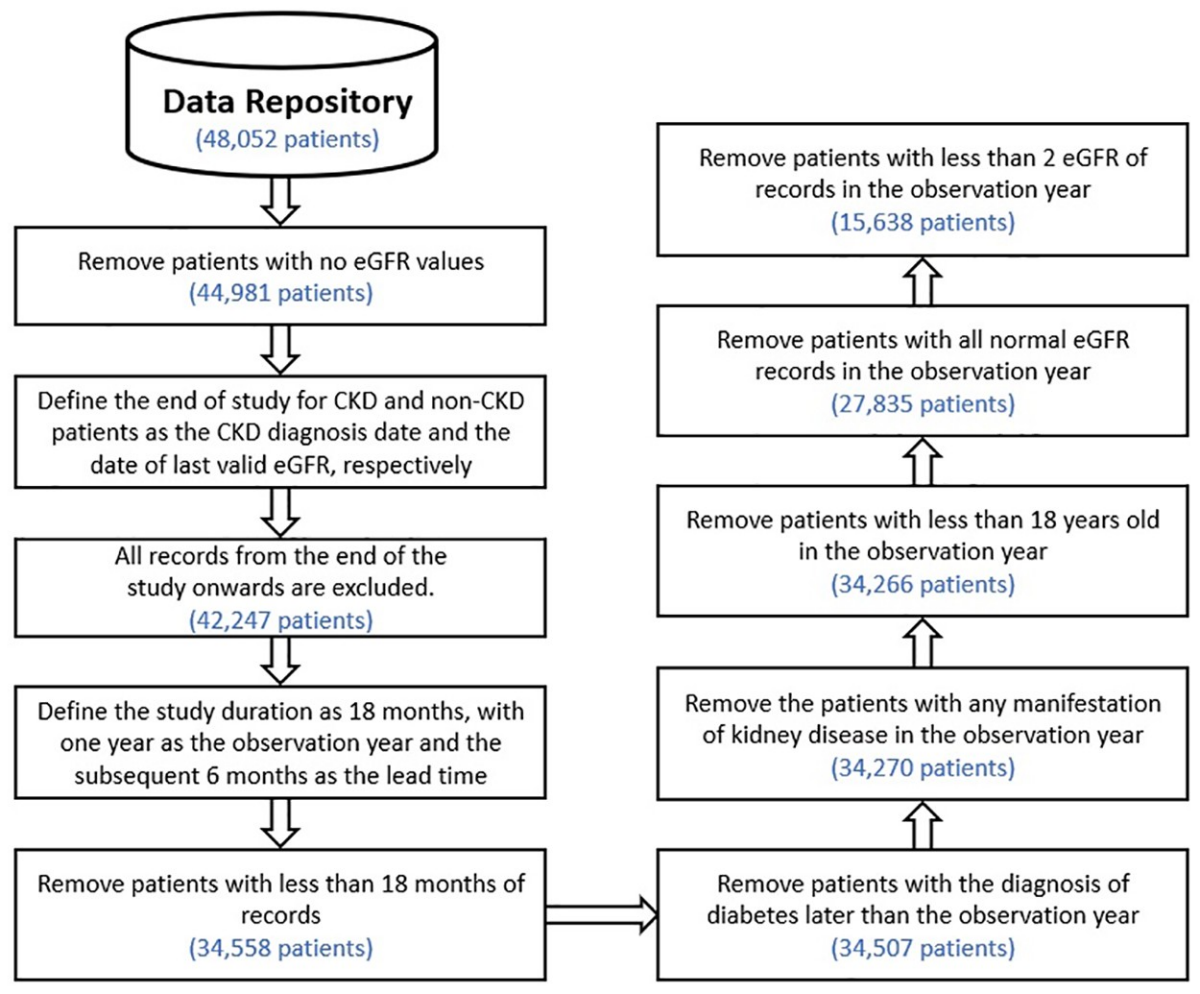
Pre-processing procedure of the data. The data is processed to exclude the patients who are not eligible for the study. After pre-processing the data of over 48, 052 diabetic patients, 15, 638 patients were found to be eligible for our model, 20.3% of which developed CKD after six months.

The end of the study for CKD patients is the CKD diagnosis date; for non-CKD patients, it is the date of the last valid eGFR record. All records from the end of the study onwards are excluded. The study duration for each patient is 18 months, with one year designated as the observation year and the subsequent 6 months as the lead time. Patients with less than 18 months of records leading up to the end of the study were excluded. The records in the lead time were removed from the data, leaving only the records in the observation year to investigate. Inclusion criteria required patients to be diagnosed with diabetes before or during the observation year and to have at least two eGFR records in the observation year. Fig 2 illustrates the steps of this phase of processing.

To address data heterogeneity in EMR data, information was aggregated within the observation year as follows:

1. The first 5 characters of the medication codes and the first 3 characters of the diagnosis codes were retained. The least frequent medication and diagnosis codes (frequency < 100 and < 10, respectively) were removed. The presence of the code in the observation year was marked as 1, otherwise as 0.
2. The diagnosis age and duration (years since the diagnosis) of 9 chronic conditions (COPD, Dementia, Depression, Diabetes, Epilepsy, Herpes Zoster, Hypertension, Osteoarthritis, and Parkinson’s) were added to the data if a patient developed it.
3. To capture the variation of each laboratory result and physical examination in the observation year, 8 summary statistics of each variable (minimum, maximum, mean, standard deviation, first quartile (Q1), second quartile (Q2), third quartile (Q3), and interquartile range (IQR)) were calculated if there were more than two records in the observation year. Otherwise, the summary statistics were replaced by null values.
4. All patients with normal eGFR values (*eGFR* > 90) in the observation year is removed from the study.
5. Demographic information of each patient (age, sex) and risk factors (smoking history, alcohol history) is added to the aggregated data. The inclusion criterion is that the patient should be at least 18 years old in the observation year.
6. The target variable is the CKD development after 6 months, which is a binary outcome: 1 for developing CKD and 0 otherwise.

**Fig 3:**
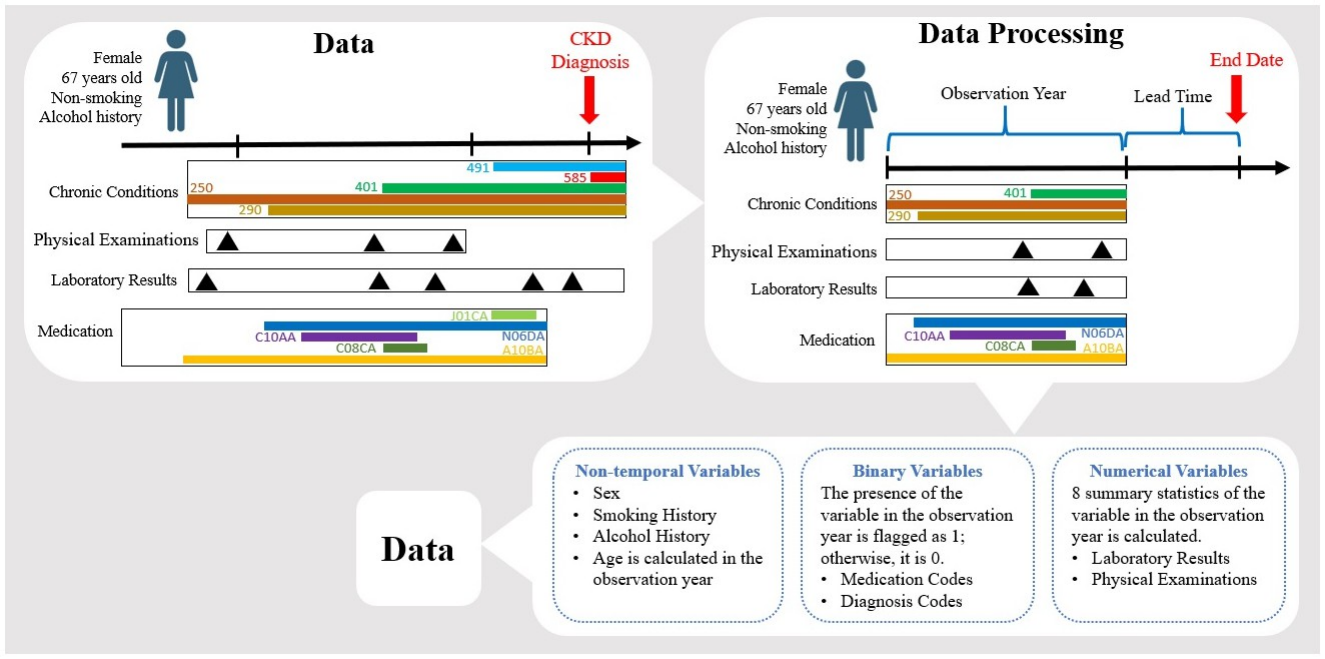
Pre-processing procedure of the data. For CKD patients, the end of the study is the date of CKD diagnosis, whereas for non-CKD patients, it is the date of the last valid eGFR. The study spans 18 months, retrospectively measured from the endpoint, with the initial 12 months as the observation year and the subsequent 6 months as the lead time. Numerical and binary variables within the observation year are aggregated using two different approaches. Non-temporal variables are subsequently incorporated into this aggregated data, which is then employed in an Xgboost model.

The pre-processed data consists of 15, 638 patients with 786 covariates and 1 target variable called the CKD flag. Fig 3 illustrates the steps involved in the pre-processing procedure.

### Proposed model

We used the Extreme Gradient Boosting model, Xgboost [18], a powerful machine learning algorithm for the prediction task. There are two main reasons we prefer Xgboost over other machine learning algorithms. First, Xgboost usually outperforms other machine learning algorithms in case of an imbalanced dataset. Second, Xgboost can treat the missing values properly, eliminating the need for missing imputation. By employing sparsity-aware split-finding techniques [18], Xgboost identifies the optimal direction to split data with missing values during the tree-building process, allowing it to handle these missing values effectively without requiring explicit imputation beforehand. Moreover, the model performed better without any imputation than using mean, median, KNN, or MICE imputation methods. As a result, we chose not to impute the data. This finding suggests that the original data, even with missing values, provided more reliable inputs for the model than the imputed datasets. Imputation, while useful in some contexts, may have introduced noise or biased estimates that negatively impacted model performance. Therefore, retaining the original data without imputation allowed the model to achieve better predictive accuracy.

To enhance the Xgboost model’s effectiveness, we focused on refining its performance through hyperparameter tuning. The optimization of Xgboost parameters aimed to strike a balance between overfitting and underfitting. This fine-tuning process was executed using the Randomized Search CV (Cross Validation) technique, randomly exploring and evaluating combinations through cross-validation to identify optimal hyperparameters, significantly enhancing the model’s performance.

The data exhibited an imbalanced distribution of positive and negative classes, with only 20.3% representing CKD patients. To handle this imbalance, we employed three strategic approaches. First, stratified *k*-fold cross-validation with *k* = 3 was used to train and assess the model, ensuring each fold maintained a proportional representation of target classes as in the entire dataset. Second, we performed random oversampling of the minority class and random undersampling of the majority class in the training set for each cross-validation, adjusting the CKD to non-CKD ratio to 1 : 2 while leaving the test set untouched. Due to missing values in the laboratory results and physical examinations, other resampling strategies were deemed unsuitable. We explored other advanced resampling techniques such as SMOTE and TomekLinks using imputed data, but the model performance was slightly lower. Finally, we assigned a higher weight (1.5, compared to the default value of 1) to the minority class in the Xgboost model, thereby increasing the penalty for misclassifications of the minority class. The evaluation of the model’s performance during each cross-validation training involved metrics such as Precision, Recall, F1-score, AUC, and AUPRC on both the training and test sets. Precision is the ratio between the True Positives (TP) and all the Positives. For our study, that would be the measure of patients we correctly identify as having CKD out of all the patients who actually have it. Recall is the measure of our model correctly identifying True Positives (TP). Thus, for all the patients who actually have CKD, recall tells us how many we correctly identified as having CKD. F1-score represents the harmonic mean between recall and precision scores. AUC and AUPRC were already explained in the result section.

## Results

### Full model with all variables

**Fig 4:**
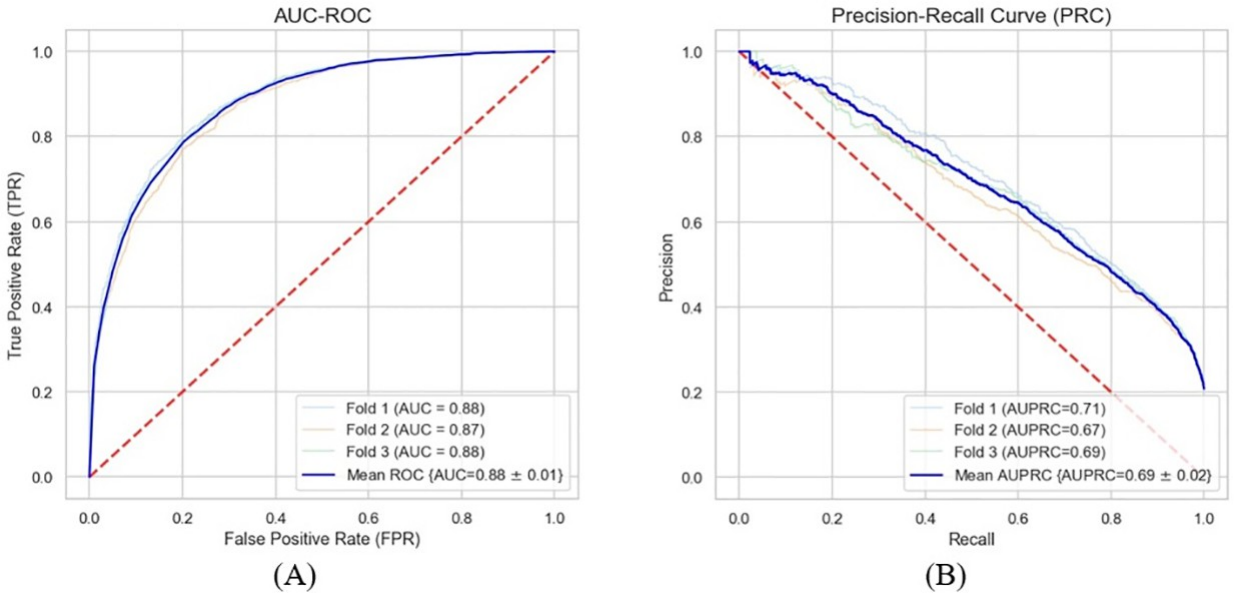
Performance of our model across three cross-validation folds. The average AUC (Area Under Receiver Operating Characteristics) of 0.88 and average AUPRC (Area Under Precision-Recall Curve) of 0.69 highlights our proposed model’s ability to distinguish between CKD and non-CKD patients.

The results demonstrate that our model can predict the onset of CKD six months in advance, achieving an average AUC (Area Under Receiver Operating Characteristics Curve) of 0.88 across three cross-validation folds. The AUC serves as a numerical summary of the ROC (Receiver Operating Characteristics) curve. The AUC is a measure of how well the model can distinguish between patients who will develop CKD and those who will not. A perfect score would be 1.0, while a score of 0.5 would indicate the model has no predictive power. An AUC of 0.88 highlights the good prediction power of our model. Additionally, we examined the Precision-Recall Curve (PRC), which captures the balance between precision and recall at different probability thresholds for a predictive model. Precision reflects the proportion of patients predicted to be at risk of CKD who actually develop the disease, while recall shows how many of the actual CKD patients were correctly identified by the model. The model’s precision was 0.55, meaning that 55% of the patients identified as high-risk actually developed CKD. The recall was 0.71, indicating that the model correctly identified 71% of all CKD cases. These results suggest that the model is reliable for early risk identification, which can guide timely interventions. As we change the decision threshold of the classifier, the precision and recall will vary, which is illustrated in Precision-Recall Curve (PRC). Typically, the precision decreases as recall increases because more false positives are included as the threshold is lowered. The ideal Precision-Recall curve is one that stays at 1.0 precision across all levels of recall, meaning the classifier makes no false positive predictions and catches all true positives. Our model demonstrated An AUPRC (Area Under Precision-Recall Curve) of 0.69, which is a reasonable ability to discriminate between CKD and non-CKD patients. Details of precision, recall, and F1-score for each fold are presented in Table 4. The model consistently performs well across all three randomly selected folds, as illustrated in Fig 4.

**Table 4:** Summary of performance of the model across three folds.

	Precision	Recall	F1-score
Fold 1	0.55	0.72	0.62
Fold 2	0.55	0.70	0.62
Fold 3	0.54	0.71	0.61
Mean	0.55	0.71	0.62

The table summarizes the performance of our proposed model across three cross-validation folds. The model performance is stable across folds with an average precision of 0.55, recall of 0.71, and F1-score of 0.62.

**Fig 5:**
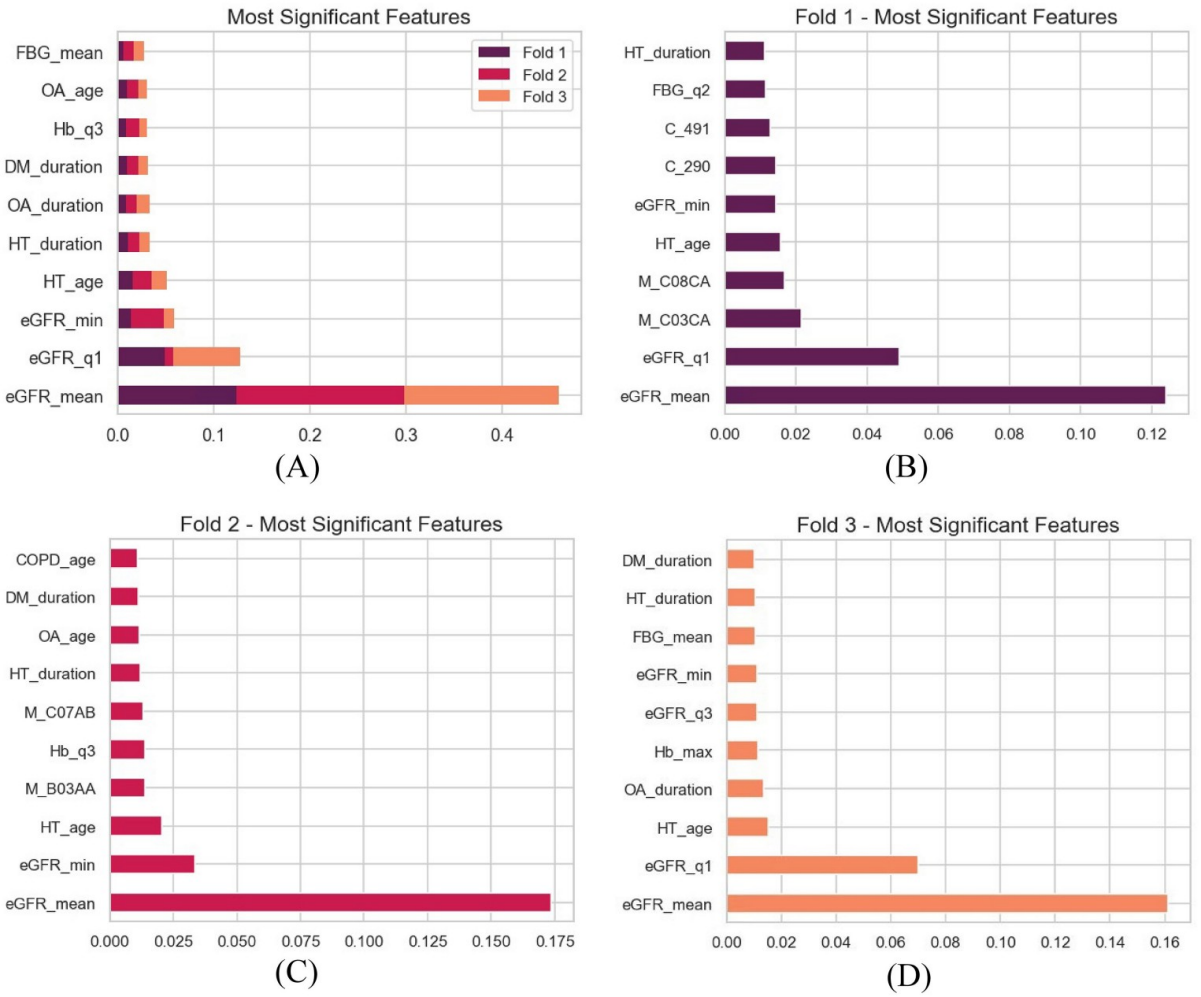
Most significant features for CKD prediction. The first figure illustrates the most significant features cumulatively across three cross-validation folds. The rest shows the details of the most significant features of each fold independently. *The variable eGFR_q1 refers to the first quartile of eGFR values in the observation year. The variables HT_age and OA_age refer to the Diagnosis age of Hypertension and Osteoarthritis, respectively. The variables HT_duration, OA_duration, and DM_duration refer to the duration of Hypertension, Osteoarthritis, and Diabetes Mellitus. The variable Hb_q3 refers to the third quartile of hemoglobin.

The most significant features of the classification task have been identified and are depicted in Fig 5. The top ten features are selected through our Xgboost feature selection process based on the normalized gain value. In Xgboost, gain refers to the reduction in loss function (or improvement in performance) that a feature contributes when it is used to split a node in a decision tree. Each normalized gain value represents the relative importance of each feature in the model and is normalized to sum to 1. A higher score for a feature indicates that it plays a more significant role in the model’s predictive performance, as it contributes more to reducing the loss function during the tree-building process. The first graph illustrates the most significant features cumulatively observed across three cross-validation folds. Among these features, the mean eGFR in the observation year is the primary factor influencing the onset of CKD, with a big difference with the next feature. This is followed by the minimum and first quartile of eGFR during the observation year. Factors such as the age at diagnosis of Hypertension and Osteoarthritis and the duration of Hypertension, Osteoarthritis, and diabetes contribute to predicting the onset of CKD. Furthermore, the third quartile of hemoglobin and the maximum fasting blood glucose (FBG) in the observation year are identified as other significant predictors.

**Fig 6:**
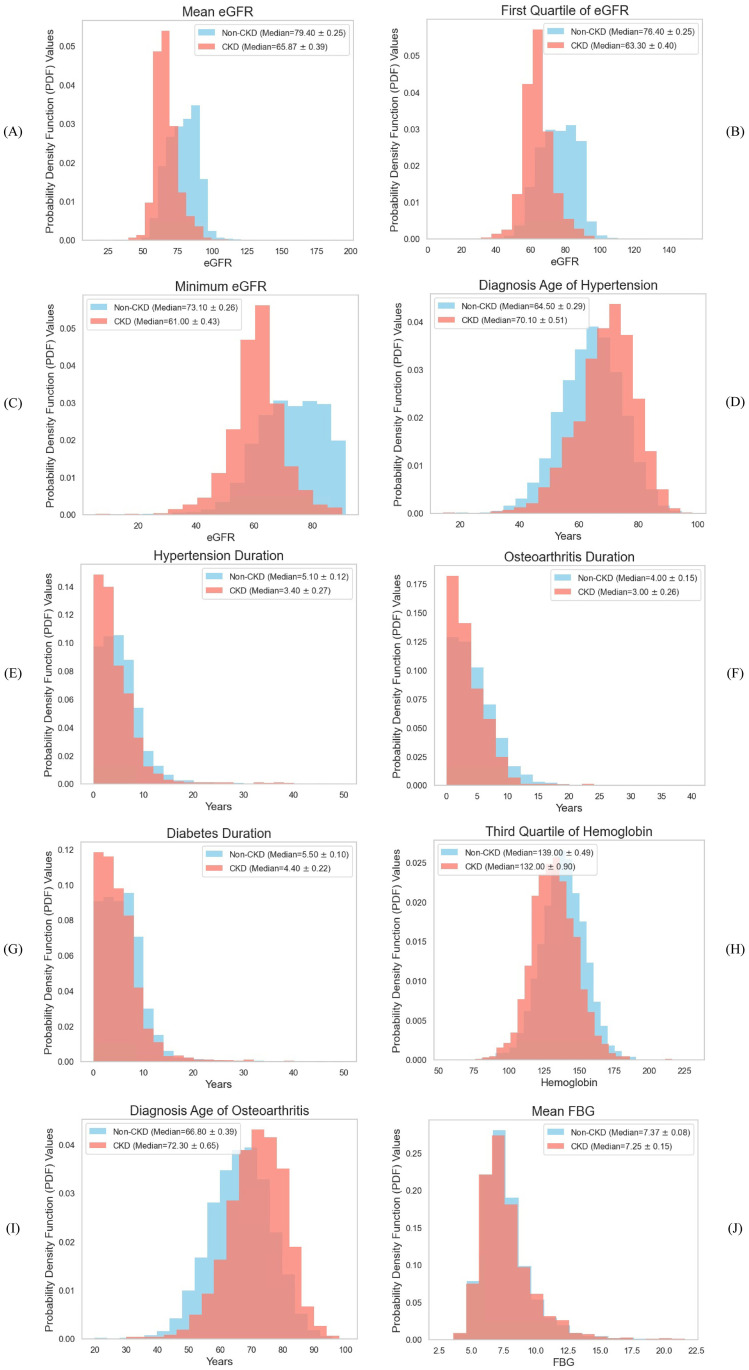
Distribution of most significant features. The histograms demonstrated the distribution of each variable for CKD and non-CKD patients, with median and 99% confidence interval.

**Fig 7:**
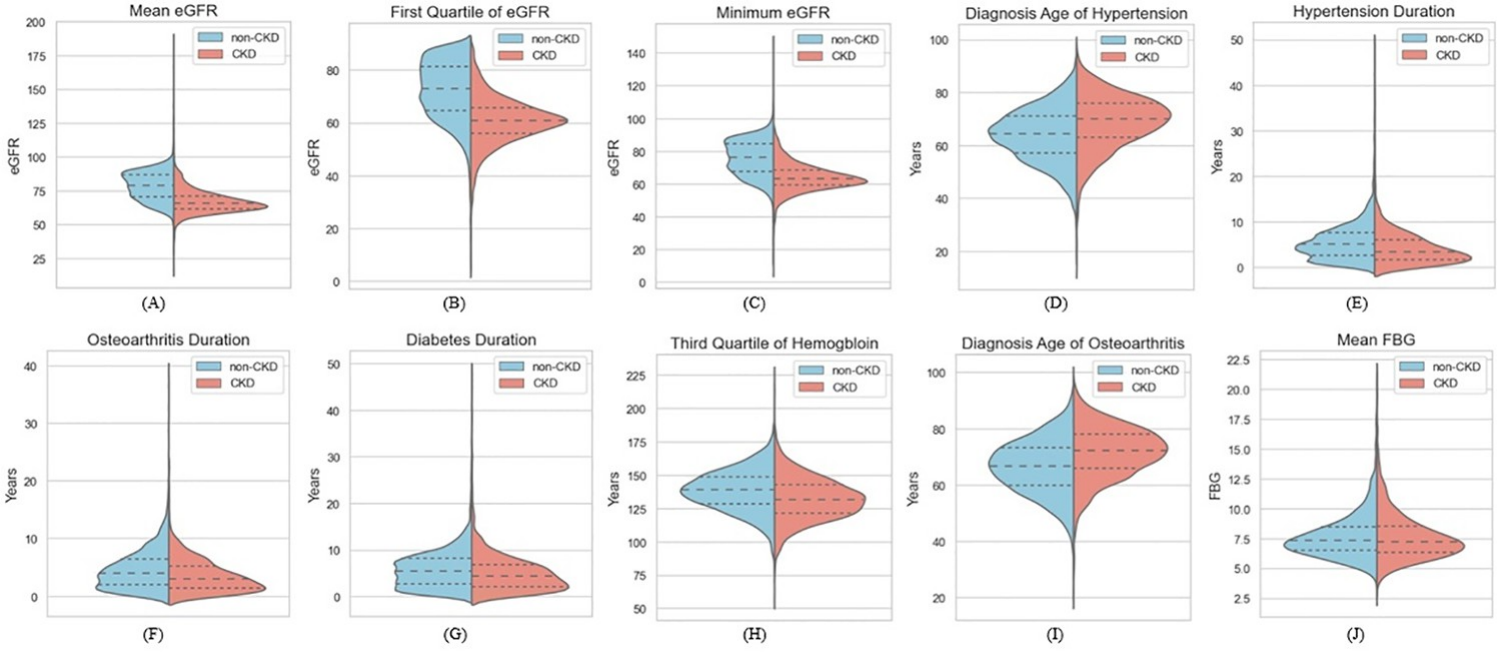
Distribution of most significant features. The Violin plots demonstrated the distribution of each variable for CKD and non-CKD patients. In each violin plot, the three dashed lines represent First Quartile (*Q*1), Median, and Third Quartile (*Q*3) from bottom to top, respectively.

Figs 6 and 7 illustrate the distribution of these most significant features as histograms (Fig 6) and violin plots (Fig 7). In the violin plots, the three dashed lines represent First Quartile (*Q*1), Median, and Third Quartile (*Q*3) from bottom to top, respectively. Mean eGFR was the most prominent feature for predicting CKD onset. CKD patients exhibited significantly lower mean eGFR values six months before diagnosis, with a median of 65.87, compared to 79.40 for non-CKD patients. The violin plot shows that 75% of CKD patients had a mean eGFR of less than 71.3, whereas only 25% of non-CKD patients had a mean eGFR below 70.9. Minimum eGFR and first quartile of eGFR followed a similar pattern, with CKD patients having lower values for both features compared to non-CKD patients. These findings reinforce the importance of reduced kidney function (as indicated by lower eGFR values) as a strong predictor of CKD. This concludes that Lower mean, minimum, and first quartile eGFR values were highly predictive of CKD onset, suggesting that patients with consistently lower kidney function are at a significantly increased risk of developing CKD.

Diagnosis age of hypertension was also a key predictive factor. CKD patients were typically diagnosed with hypertension later in life, with a median diagnosis age of 70.1 years, compared to 64.5 years for non-CKD patients. This suggests that late-onset hypertension may accelerate CKD progression. Duration of hypertension was shorter in CKD patients (median of 3.40 years) compared to non-CKD patients (5.10 years). This finding suggests that a later onset of hypertension, even with a shorter duration, may be more detrimental to kidney health. Both results indicate that individuals diagnosed with hypertension later in life are at higher risk of CKD development, highlighting the importance of early management of hypertension, particularly in older populations.

The diagnosis age of osteoarthritis was significantly higher in CKD patients than non-CKD patients (*p* − *value* < 0.01). CKD patients also had shorter durations of osteoarthritis and diabetes (*p* − *value* < 0.01), suggesting that developing these conditions later in life might be associated with an increased risk of CKD. Older age at the onset of osteoarthritis, shorter durations of osteoarthritis, and diabetes appear to be a risk factor for CKD. This may be due to the increased vulnerability of older patients to comorbid conditions that contribute to kidney disease.

The third quartile of hemoglobin levels was lower in CKD patients than in non-CKD patients, indicating that anemia is more prevalent among CKD patients. This aligns with the known relationship between anemia and declining kidney function. Mean FBG did not significantly differ between CKD and non-CKD patients (*p* − *value* = 0.71), suggesting that glycemic control (as measured by FBG) was not a major differentiator in CKD risk in this diabetic cohort. In conclusion, hemoglobin levels, particularly in the third quartile, were important for predicting CKD, while FBG was not a significant contributor, likely due to the overall management of blood glucose in the diabetic population studied. Table 5 presents the mean value of each significant feature for the two groups.

**Table 5:** Mean value of CKD and non-CKD patients.

	CKD	non-CKD	p-value
Mean eGFR	67.23	78.99	< 0.01
Q1 of eGFR	64.23	75.96	< 0.01
Minimum eGFR	60.84	72.37	< 0.01
Hypertension Diagnosis Age	69.04	64.07	< 0.01
Hypertension Duration	4.52	5.60	< 0.01
Osteoarthritis Duration	3.61	4.68	< 0.01
Diabetes Duration	5.13	5.94	< 0.01
Q3 of Hemoglobin	132.34	138.68	< 0.01
Osteoarthritis Diagnosis Age	71.66	66.54	< 0.01
Mean FBG	7.70	7.72	0.71

The table illustrates the mean of the most significant features for CKD and non-CKD patients. All of the variables except the mean FBG are significantly different (*p*-value < 0.01) for the two groups.

### Model with selected variables

The baseline model with only sex, age, and 8 measures of eGFR in the observation year demonstrated an average AUC of 0.78 and an average AUPRC of 0.44 over three cross-validation folds. Despite the mean eGFR in the observation year being the predominant feature in the full model for the prediction task, its diminished predictive power compared to the full model suggests that relying solely on eGFR values is inadequate for predicting CKD incidence in advance.

On the other hand, the model with the 10 most significant features demonstrated performance akin to the full model, albeit with slightly lower AUC And AUPRC. This model illustrated an AUC of 0.86 and AUPRC of 0.65 over three cross-validation folds, indicating our model’s generalizability. The full model with more than 700 variables might not be feasible for various populations, but focusing on only the most significant features would give clinicians the flexibility to assess the risk of CKD for each patient. This might be helpful for clinicians to consider other variables besides eGFR, especially chronic conditions. Notably, if a patient develops hypertension, osteoarthritis, and diabetes in the older ages, greater attention must be paid to kidney function in order to reduce the occurrence of CKD. The results for three models are summarized in the Table 6.

**Table 6:** Summary of performance of the three models.

	AUC	AUPRC	Precision	Recall	F1-score
Baseline model with eGFR values	0.78	0.44	0.42	0.64	0.51
Full model with all variables	0.88	0.69	0.55	0.71	0.62
Model with most significant features	0.86	0.65	0.51	0.72	0.60

The table summarizes the performance of the model with three different inputs. The first model is the full model with all variables. The baseline model takes only age, sex, and 8 measurements of eGFR values as input. The last model takes the most significant features identified in the full model as the input.

## Discussion

In this study, we have implemented a prognostic model for predicting CKD onset six months in advance in diabetic patients showing signs of kidney function impairment. Our model utilized 786 variables in the observation year and achieved an average AUC of 0.88 and AUPRC of 0.69 in three cross-validation folds, showing good discriminatory performance. Our study demonstrated that lower mean, minimum, and first quartile of eGFR values in the observation year, developing hypertension, diabetes, and osteoarthritis in the later stages of life, and lower third quartile of hemoglobin in the observation year are associated with CKD progression in diabetic patients.

To investigate the generalizability of our model, we explored two additional models, the first one as the baseline model only with sex, age, and eGFR summary statistics in the observation year and the second model with the 10 most significant features from the full model. Even though the mean eGFR was the first most significant variable with a big difference compared to the next one in the full model, the model with only eGFR values demonstrated lower performance power, with AUC of 0.78 and AUPRC of 0.44. In contrast, the model with the most significant features illustrated a slightly lower performance than the full model with an AUC of 0.86 and AUPRC of 0.65. This reflects the influence of comorbidities on CKD progression besides eGFR values. Comorbidities such as diabetes, hypertension, and osteoarthritis as risk factors are potentially controllable, and the importance of their control should be emphasized in patient education and clinical management. Predicting CKD one year and two years in advance led to decreased model performance. The AUC and AUPRC for the one-year prediction were 0.83 and 0.59, respectively, while for the two-year prediction, they were 0.81 and 0.54. This suggests that eGFR may begin to decline relatively shortly before CKD onset, warranting further investigation.

Recently, various predictive models have been established to predict the progression of CKD. Huang et al. [19] predicted CKD development in the next 6.5 years from metabolites and clinical variables with machine learning models and achieved an AUC of 0.857. Makino et al. [20] utilized Natural Language Processing (NLP) and time series deep learning models to predict the progression of CKD from stage 1 to stages 2-5 in the next 6 months with AUC of 0.74. Su et al. [21] predicted the onset of CKD in elderly diabetic patients in China using panel data with six machine-learning models and achieved the highest AUC of 0.89, similar to our results. In this study, the data included self-reported daily activity beside EHR data, which is unavailable in our study. Krishnamurthy et al. [22] developed a machine-learning model that uses the comorbidities information and medication data of 90, 000 patients to predict the CKD onset within the next 6 or 12 months and achieved AUC of 0.957 and 0.954, respectively. This study used temporal quarterly and monthly aggregated data from their respective two-year observation period as input for the Convolutional Neural Networks (CNN) model. Our study aggregated the data at the year-level due to the lack of a sufficiently big dataset. In the future, upon sufficient data, the month-level and quarter-level aggregation can be investigated in our model. Nelson et al. [23] conducted an individual-level data analysis of 34 multinational cohorts from the CKD Prognosis Consortium, including 5, 222, 711 individuals in 34 multinational cohorts from 28 countries from 1970 to 2017. The model for the prediction task was developed from weighted-average hazard ratios estimated in all participating cohorts and an adjusted baseline risk estimated in cohorts with frequent outcome assessment. The risk models had a median AUC for the 5-year predicted probability of 0.845 in the cohorts without diabetes and 0.801 in the cohorts with diabetes. Gurudas et al. [24] used a primary care dataset of 20,510 multi-ethnic individuals with T2DM from London, UK, and developed models to predict 5-year CKD risk using Cox proportional hazards regression and achieved AUC of 0.85. Chien et al. [25] developed a point-based model to predict the 4-year risk of incident CKD in a Chinese population according to five variables: age, body mass index, diastolic blood pressure, and history of type 2 diabetes, and stroke, uric acid, postprandial glucose, hemoglobin A1c, and proteinuria 100 mg/dL or greater. The model, built using a multivariate Cox proportional hazards approach combined with a categorization point system, achieved an AUC of 0.765 to 0.768. There are also various studies using traditional statistical methods to predict CKD incidence in advance, such as [26–29] with the model performance of AUC between 0.72 to 0.85.

In comparison to the existing studies, our study has several strengths. First, our cohorts were constructed using primary care data, thus reflecting the information typically available. One of the challenges with EMR data is the heterogeneity of the data. By aggregating the data in the observation year, we overcame some of the challenges faced in using primary care data, particularly longitudinal EMR data, including irregularly sampled data and varying lengths of patient history. To capture the variation of the numerical variables in the observation year, we calculated summary statistics of each numerical variable for the observation year. We also calculated the duration and diagnosis age for 9 chronic conditions in the observation year. For chronic condition diagnosis codes and medication codes, we aggregated them as the presence of absence in the observation year. This aggregating strategy allowed us to integrate many data resources. Furthermore, creating risk prediction models specific to countries or territories enables the integration of local population-based factors and clinician management approaches, ensuring more accurate risk prognostication for the local population. Specifically, considering variations in race, lifestyle, and geographical conditions, there is a continued need to formulate a reliable predictive model for chronic kidney disease (CKD) in diverse ethnic groups. This can contribute to the early identification of individuals with elevated CKD risks. We identified significant features such as eGFR, diagnosis age and duration of specific chronic diseases (osteoarthritis, hypertension, diabetes), Hemoglobin, and FBG. Healthcare professionals can use these features to categorize patients at higher risk of CKD development, allowing for early intervention. Notably, our findings emphasize that older individuals face an increased risk of CKD development when they develop hypertension, osteoarthritis, or diabetes, highlighting the importance of identifying and implementing preventive measures for such patients to enhance their quality of life. Moreover, One of the main challenges in healthcare data is that the data is imbalanced in favor of negative cases. This usually affects the prediction power since the model is more biased toward the negative class. In addressing the challenge of imbalanced healthcare data, we employed three strategies: stratified cross-validation, undersampling and oversampling, and adjustment of loss function weights, leading to improved prediction power.

There are several limitations in this study. First, our sample size was moderate in comparison with other studies. As this research study utilized data from Canada, its scope was confined by geographical and demographic limitations, potentially introducing biases related to the population’s specific characteristics. Consequently, the model’s generalizability to the global population or other regions is restricted and must be further investigated in the future. The lack of external validation is a significant limitation that must be addressed in future research. The model’s applicability remains uncertain without testing it on data from different regions or among diverse populations. Additionally, data noise stemming from human and technical errors, which can be challenging to detect, might further impact the model’s effectiveness. This model does not include BUN and ACR records that are essential in CKD diagnosis in addition to eGFR. This exclusion, driven by the unavailability of these variables in many cases, may have introduced bias and impacted the model’s predictive power. In the future, the model performance can be investigated in the presence of these two variables. Socioeconomic factors such as income, education, and employment status were unavailable in this research study, and considering the impact of the socioeconomic factors might be valuable sources of information for CKD prediction and can be investigated in future work. Furthermore, other influential factors, such as genetic factors, diet, exercise, and lifestyle, were not considered in our study and warrant exploration in future research. In terms of clinical implications, while our model shows promise in improving CKD risk evaluation, its integration into clinical practice requires careful consideration. The lack of external validation limits our ability to predict how the model will perform in real-world clinical settings. Therefore, evaluating the model’s impact on patient outcomes in future clinical practice is essential to ensure its applicability. Additionally, the response of clinicians to using machine learning models remains uncertain, and future studies should explore how the model can be integrated into clinical workflows to improve the management of patients at risk of CKD. In conclusion, while our model has the potential to advance CKD management, further research is necessary to validate its effectiveness across different populations and settings. This includes incorporating data from other regions, assessing the impact of socioeconomic factors, and evaluating the model’s integration into clinical practice to ensure that it can effectively improve patient outcomes.

## Conclusion

In conclusion, we developed a machine-learning model to predict the occurrence of chronic kidney disease (CKD) in diabetic patients showing signs of kidney function impairment in the next six months, achieving a discrimination capability with an average AUC of 0.88 across three cross-validation folds. The model utilizes routinely collected healthcare data, including demographics, laboratory results, medications, and chronic conditions, enabling efficient early detection of CKD. Analysis of 786 variables revealed that the mean, minimum, and first quartile of eGFR in the observation year, along with the diagnosis age of hypertension and osteoarthritis, duration of hypertension, osteoarthritis, and diabetes, as well as the third quartile of hemoglobin and mean of FBG in the observation year, influenced the risk of CKD onset. Additionally, we explored a model based on these most significant features, achieving slightly lower results than the full model, with an AUC of 0.86. With only a few clinically available variables, this model holds the potential for deployment in healthcare to predict CKD onset in advance. Such models could assist in real-time risk assessment and targeting preventive interventions for individuals at higher risk of developing CKD, ultimately enhancing their quality of life.
